# Knock-In Mouse Models to Investigate the Functions of Opioid Receptors *in vivo*

**DOI:** 10.3389/fncel.2022.807549

**Published:** 2022-01-31

**Authors:** Jade Degrandmaison, Samuel Rochon-Haché, Jean-Luc Parent, Louis Gendron

**Affiliations:** ^1^Centre de Recherche du Centre Hospitalier Universitaire de Sherbrooke, Département de Médecine, Institut de Pharmacologie de Sherbrooke, Faculté de Médecine et des Sciences de la Santé, Université de Sherbrooke, Sherbrooke, QC, Canada; ^2^Centre de Recherche du Centre Hospitalier Universitaire de Sherbrooke, Département de Pharmacologie-Physiologie, Institut de Pharmacologie de Sherbrooke, Faculté de Médecine et des Sciences de la Santé, Université de Sherbrooke, Sherbrooke, QC, Canada; ^3^Quebec Network of Junior Pain Investigators, Sherbrooke, QC, Canada; ^4^Quebec Pain Research Network, Sherbrooke, QC, Canada

**Keywords:** G protein-coupled receptor (GPCR), opioid receptor, knock-in (KI) mice, nociceptin receptor, mouse model, fluorescent protein, *in vivo*

## Abstract

Due to their low expression levels, complex multi-pass transmembrane structure, and the current lack of highly specific antibodies, the assessment of endogenous G protein-coupled receptors (GPCRs) remains challenging. While most of the research regarding their functions was performed in heterologous systems overexpressing the receptor, recent advances in genetic engineering methods have allowed the generation of several unique mouse models. These animals proved to be useful to investigate numerous aspects underlying the physiological functions of GPCRs, including their endogenous expression, distribution, interactome, and trafficking processes. Given their significant pharmacological importance and central roles in the nervous system, opioid peptide receptors (OPr) are often referred to as prototypical receptors for the study of GPCR regulatory mechanisms. Although only a few GPCR knock-in mouse lines have thus far been generated, OPr are strikingly well represented with over 20 different knock-in models, more than half of which were developed within the last 5 years. In this review, we describe the arsenal of OPr (mu-, delta-, and kappa-opioid), as well as the opioid-related nociceptin/orphanin FQ (NOP) receptor knock-in mouse models that have been generated over the past years. We further highlight the invaluable contribution of such models to our understanding of the *in vivo* mechanisms underlying the regulation of OPr, which could be conceivably transposed to any other GPCR, as well as the limitations, future perspectives, and possibilities enabled by such tools.

## Introduction

Characterized by a common topology exhibiting an extracellular *N*-terminal domain, seven hydrophobic membrane α-helices and a cytosolic *C*-terminus, G protein-coupled receptors (GPCR) form the largest family of transmembrane proteins (Hauser et al., 2017). With over 800 different members, these receptors can respond to a vast array of ligands, including peptides, lipids, photons, neurotransmitters, and hormones to fine-tune virtually every physiological system (Hauser et al., 2017). Most importantly, GPCRs represent long-standing powerful therapeutic targets with approximately 34% of the currently marketed drugs targeting these transmembrane proteins (Hauser et al., 2017; Kooistra et al., 2021). Despite their significant clinical importance, the study of GPCRs *in vivo* has been impaired by several challenges. Indeed, their relatively low endogenous expression levels, their complex transmembrane structure and the lack of specific and potent antibodies have contributed to limit our understanding of their functions in physiologically relevant conditions (Michel et al., 2009; Jo and Jung, 2016). The design of new tools and approaches to study GPCRs *in vivo* is therefore crucial to develop and improve current therapeutics.

Belonging to the class-A GPCRs, the opioid receptors family (OPr), which comprises the mu (μ, MOPr), delta (δ, DOPr) and kappa (κ, KOPr) subtypes, share between 59 and 63% of amino acids sequence identity (Pathan and Williams, 2012; Degrandmaison et al., 2021). Moreover, the nociceptin receptor (NOPr), originally referred to as the κ-type 3 opioid receptor, is also closely related to the OPr family (Borsodi et al., 2019). Opioid and nociceptin receptors are predominantly expressed throughout the nervous system where they specifically regulate a wide range of physiological effects, mostly associated with the nociception-, stress-, mood-, and reward-related pathways. Although the pharmacological profile exhibited by the NOPr is relatively more complex (discussed in section “Nociceptin/orphanin FQ receptor knock-in mouse lines”), ligand-mediated activation of these receptors results in analgesia, as well as other distinct physiological responses (Reinscheid et al., 1995; Grisel et al., 1996; Mogil et al., 1996a,b; Xu et al., 1996; Yamamoto et al., 1997; Mogil and Pasternak, 2001; Borsodi et al., 2019). Given their significant clinical relevance, OPr are established as a model family of receptors to investigate the cellular mechanisms underlying the regulation of GPCRs, therefore placing them at the forefront of recent advances in the GPCR field.

As for other members of the GPCR family, initial research conducted on OPr and their endogenous ligands relied on the partial deletion of targeted genes within an animal, generating knock-out (KO) models. This “loss-of-function” genetic approach has revolutionized the study of opioid functions *in vivo* by offering a complementary approach to classical pharmacology. The precise deletion of each component of the endogenous opioid and NOPr-N/OFQ systems has indeed shed new light on their specific individual contribution to pathophysiological states such as acute and chronic pain (see Kieffer, 1999; Dierich and Kieffer, 2004; Nadal et al., 2013; Maldonado et al., 2018 for reviews). Further progress regarding genetically engineered mice has then led to the development of transgenic mouse lines, which are characterized by the rather random integration of an exogenous gene sequence (Doyle et al., 2012; Ceredig and Massotte, 2015). Although these models proved themselves useful for specific applications, several drawbacks have been associated to such an approach, including the overexpression of the encoded protein as compared to wild-type (WT) animals and the improper insertion of the transgene (e.g., within undesired tissue or genomic regions) (Doyle et al., 2012; Ceredig and Massotte, 2015). In recent years, an alternative strategy aiming to specifically introduce or modify a gene of interest has emerged. The generation of knock-in (KI) mouse strains consists of specifically inserting or modifying a gene at the locus of interest, thus overcoming most of the disadvantages associated with transgenic models. Such a strategy further opened the path to a myriad of possibilities regarding the creation of unique mouse lines. The study of OPr has also significantly benefited from this expanding technology as demonstrated by the 13 novel OPr KI mouse strains that have been generated between 2019-2021, for a total of more than 20 different OPr and NOPr KI mice published as of to date. These OPr-based KI mice have been designed according to diverse strategies including the fusion of the selected receptor with a fluorescent protein (FP) or a small epitope-tag [e.g., hemagglutinin (HA) or FLAG], as well as the insertion of a Cre recombinase. In the following sections, we describe the currently available KI mouse lines that have been created for NOPr and each OPr subtypes, as well as their contribution to our understanding of the endogenous molecular and physiological roles of the opioid and nociceptin systems. We also further discuss the limitations and the perspectives related to the study of OPr in animal models.

### μ-Opioid Receptor Knock-In Mouse Lines

Undoubtedly the most extensively studied opioid receptor, the MOPr is the primary target of most clinical opioid therapeutics, including morphine, fentanyl and codeine (Pathan and Williams, 2012). Although activation of the MOPr has been associated with yet unmatched analgesic properties, its agonists are also accountable for a broad range of serious adverse effects such as tolerance, dependence, addiction, constipation, and respiratory depression when used over an extended period of time (Morgan and Christie, 2011). Current research efforts on the MOPr therefore mainly focus on deciphering the cellular and physiological mechanisms involved in the development of such adverse effects, as well as the design of better-tolerated analgesics for chronic pain management. In recent years, a wide variety of MOPr KI mouse lines has been generated. As described below, these animals have provided useful insights regarding the regulation and the functions of this major pharmacological target.

**FIGURE 1:**
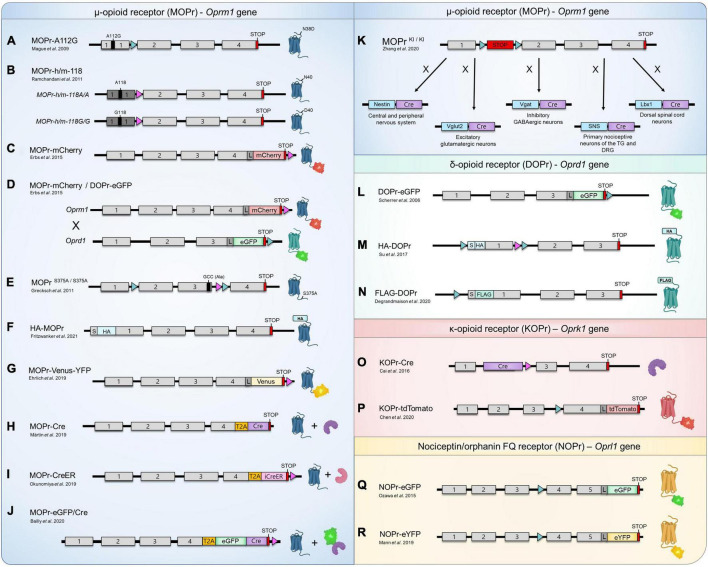
Opioid and nociceptin receptors knock-in mouse lines. **(A–R)** Schematic representation of the design of currently available KI mouse models for the study of MOPr (blue), DOPr (green), KOPr (red), and NOPr (yellow). The sequence corresponding to the mouse (gray boxes) or human (dark gray boxes) exons, START codons (S), STOP codons (red boxes), loxP sites (aqua triangles), FRT sites (pink triangles), as well as the sequences encoding for various fluorescent proteins (eGFP, mCherry, tdTomato, Venus and eYFP), epitope-tag sequences (FLAG, HA), linker (L; encoding for Gly-Ser-Ile-Ala-Thr), Cre recombinase (Cre, purple boxes), inducible Cre recombinase-modified estrogen receptor (iCreeER, pink box) and the *Thosea asigna* virus 2A-like peptide (T2A, orange boxes) are identified. The schematic representation is not to scale. (*K*) Cre-mediated recombination can also occur in other regions than those indicated.

More than a decade ago, Mague et al. (2009) have designed a MOPr KI mouse line harboring the A112G mutation, corresponding to a single nucleotide polymorphism (SNP) previously identified in the human MOPr (A118G in humans). With a prevalence of 15–30% in Europeans and 49–60% in individuals of Asian ancestry, this common point mutation resulting in the replacement of an asparagine for an aspartic acid (N40D in humans) at a potential *N*-glycosylation site has been associated with altered responses to opioid-induced analgesia, as well as to distinct phenotypes related to opioid, alcohol and nicotine addictions (Lerman et al., 2004; Ray and Hutchison, 2004, 2007; Kreek et al., 2005; Chou et al., 2006; Drakenberg et al., 2006; van den Wildenberg et al., 2007; Anton et al., 2008; Sia et al., 2008; Mague et al., 2009; Huang et al., 2012). Since several discrepancies had been observed while investigating the MOPr-N40D variant in heterologous expression systems, the authors used homologous recombination to specifically introduce the A112G mutation within the exon 1 of the *Oprm1* gene, resulting in a mouse line expressing the corresponding mutated MOPr-N38D (Figure 1A; Mague et al., 2009). Behavioral assays performed indicated that animals presenting the G112 allele exhibited reduced MOPr expression (mRNA and protein levels), as well as a reduction of the antinociceptive and hyperlocomotor effects induced by acute morphine treatment (Table 1; Mague et al., 2009). Interestingly, the authors also reported sex-specific reductions of the morphine-mediated rewarding properties and the aversive components of naloxone-precipitated morphine withdrawal with only females demonstrating an altered behavioral response (Mague et al., 2009). In a following study carried out by Wang et al. (2012), binding of the radiolabeled MOPr agonist [^3^H]-DAMGO revealed that MOPr-A112G expression is reduced in several, but not all, brain regions as compared to WT MOPr (Table 1). Whereas no significant difference in the binding of [^3^H]-DAMGO in the caudate putamen (CPu) and hippocampus was measured, higher expression of MOPr was observed in the cingulate, motor and insular cortices, amygdala, nucleus accumbens (NAc) core and shell, periaqueductal gray (PAG), ventral tegmental area (VTA) and thalamus of A/A mice (Wang et al., 2012). While most of these regions (i.e., amygdala, anterior cingulate, and insular cortices) are often referred to as the “pain matrix” of the brain, others, including the PAG, are known to be directly involved in opioid antinociception (Yaksh and Rudy, 1976; Ingvar, 1999; Brooks and Tracey, 2005). These results are therefore consistent with behavioral experiments indicating that mice carrying the G112 allele present a reduced antinociceptive response to morphine when compared to A/A mice (Mague et al., 2009; Wang et al., 2012).

**TABLE 1:** Characterization of the expression levels, subcellular localization and internalization of genetically modified opioid receptors from KI mice.

KI mouse line	Receptor density (*vs.* WT or CTRL mice)	Subcellular localization and internalization	References
MOPr-A112G	Lower in G/G animals	N/A	Mague et al., 2009; Wang et al., 2012
MOPr-h/m-118G/G, MOPr-h/m-118A/A	Similar between G/G and A/A mice	N/A	Ramchandani et al., 2011
MOPr-mCherry	Slightly higher	Predominant intracellular localization. DAMGO-mediated internalization is detectable in primary hippocampal neurons	Erbs et al., 2015
MOPr-Cre and MOPr-CreER	N/A	N/A	Märtin et al., 2019; Okunomiya et al., 2020
MOPr-eGFP-Cre	N/A, but similar mRNA levels	N/A	Bailly et al., 2020
HA-MOPr	Lower (∼50% less in KI mice)	N/A	Fritzwanker et al., 2021
MOPr-Venus-YFP	N/A, but similar mRNA levels	Internalization of MOPr-Venus-YFP in DRG neurons following treatments with DAMGO and Met-Enk, but not buprenorphine nor PZM21.	Ehrlich et al., 2019
MOPr *^S375A/S375A^*	Similar	Similar subcellular distribution as WT.	Grecksch et al., 2011
DOPr-eGFP	Higher (∼1.5-fold)	Predominant PM localization. SNC80 induces endocytosis *in vivo* and in primary CPu neurons.	Scherrer et al., 2006
HA-DOPr	N/A	N/A	Su et al., 2017
FLAG-DOPr	Similar	N/A	Degrandmaison et al., 2020
KOPr-Cre	N/A	N/A	Cai et al., 2016
KOPr-tdTomato	Higher (∼12-fold)	U50,488 treatment induces translocation of KOPr-tdT from PM to intracellular space.	Chen et al., 2020
NOPr-eGFP	Higher (∼2.8-fold)	Predominant intracellular localization, but some PM-localized NOPr are also observed. N/OFQ mediates internalization in primary neurons*.	Ozawa et al., 2018
NOPr-eYFP	N/A	*In vivo* internalization in primary neurons with various compounds including agonist N/OFQ.	Mann et al., 2019

CPu, caudate putamen; CTRL, control; DRG, dorsal root ganglion; PM, plasma membrane; N/A, not assessed; WT, wildtype. *Not clearly evident due to the weak expression of NOPr-eGFP at the neuronal PM.

It has been suggested that the region-specific differences in MOPr expression levels between A112 and G112 mice might be attributed to the variation in *N*-glycan types and contents across the brain (Wang et al., 2012). As reported previously, *N*-glycosylation of GPCRs, a post-translational modification (PTM) mainly occurring in the ER and Golgi apparatus, plays an important role in the correct folding and maturation of the receptors, thereby impacting their neuronal membrane density (Goth et al., 2020; Lemos Duarte and Devi, 2020; Degrandmaison et al., 2021). Presumably due to the differential expression of glycosyltransferases throughout the mouse brain, *N*-glycosylation of MOPr has been shown to be variable depending on the brain areas (Matsuhashi et al., 2003; Huang et al., 2008, 2012). For example, Huang et al. (2008) have observed that the WT MOPr is differentially modified by *N*-glycans in the CPu vs. the thalamus of the rat and mouse, thus suggesting brain region-specific *N*-glycosylation patterns. In a follow-up study, the authors indicated that the A112G mutation (or A118G in human MOPr) led to a reduction in MOPr *N*-glycosylation in the mouse brain and in cultured cells, which highlights the asparagine 38 (or 40) of the MOPr as a probable glycosylation site (Huang et al., 2012). Since sex-differences in *N*-linked glycosylation have been already reported, with males having higher levels of several types of *N*-glycans, this hypothesis could also possibly explain the results obtained for the variations in MOPr expression levels (Knežević et al., 2009; Stanta et al., 2010). Other factors, such as epigenetic regulation and the hormonal state of the females, might represent possible causes for this sexual dimorphism (Wang et al., 2012).

A few years later, the involvement of this specific polymorphism in alcohol use disorders was further investigated by Ramchandani et al. (2011). In this study, the authors created two humanized KI mice in which the *Oprm1* first exon was replaced by the corresponding human sequence in order to generate homozygous mouse lines carrying each one of the variants (i.e., G/G or A/A) (Ramchandani et al., 2011; Figure 1B). These mice, henceforth referred to as MOPr-h/m-118A/A (major 118A allele) and MOPr-h/m-118G/G (minor 118G allele, corresponding to the SNP) showed distinctive striatal dopamine responses when submitted to an alcohol challenge, thus suggesting that the A118G variation represents a genetic determinant possibly modulating alcohol reward (Ramchandani et al., 2011). Interestingly, as opposed to the results reported by Wang et al. (2012) no differences in [^3^H]-DAMGO binding have been observed in the dorsal striatum, NAc and VTA of G118 and A118 mice (Ramchandani et al., 2011; Table 1). Although it was suggested that the replacement of the *Oprm1* exon 1 with the homologous human sequence could affect MOPr protein expression and/or maturation, the exact cause explaining this discrepancy remains to be investigated.

Erbs et al. (2015) have generated a MOPr-mCherry KI mouse line. The design used homologous recombination to replace the *Oprm1* STOP codon with the sequence encoding the gene for the red protein mCherry followed by an FRT flanked neomycin resistance gene. The resistance gene was then excised by a FLP recombinase targeting the FRT sites, resulting in a mouse expressing MOPr fused with mCherry at its *C*-terminus (Figure 1C). In a concomitant study, the same group mapped the expression of MOPr-mCherry within the habenular complex, revealing a strong expression of the receptor in the medial habenula (MHb), *fasciculus retroflexus* and interpeduncular nucleus (IPN) (Gardon et al., 2014). Since the MHb has been previously recognized as a region mediating the analgesic and rewarding properties of opioids, the expression of MOPr in several compartments of the MHb-IPN axis provided important insights regarding its physiological functions (Gardon et al., 2014). The presence of MOPr in astrocytes of the VTA, NAc and in the CA1 area of the hippocampus has also been observed using these mice (Nam et al., 2018). The MOPr-mCherry KI mouse line has also been used by Mambretti et al. (2016) to analyze the expression, localization, and potential functions of MOPr along nociceptive axons under physiological conditions. Through combination of immunostaining experiments and immunoelectron microscopy, the study showed that the receptor is present in the cytoplasm and membrane of unmyelinated axons from the sciatic nerve (Mambretti et al., 2016). While perisciatic injection of the lipophilic MOPr agonist fentanyl increased nociceptive thresholds, simultaneous co-administration with its antagonist naloxone completely reversed this physiological response, thereby confirming that axonal MOPr are functional in naïve animals (Mambretti et al., 2016).

Erbs et al. (2015) thereafter generated a double knock-in (dKI) mouse line by breeding MOPr-mCherry mice with DOPr-eGFP (enhanced Green Fluorescent Protein) mice (Figure 1D). In this elegant and original study, the authors have mapped the distribution of both opioid receptors throughout the nervous system and provided the results as an interactive database allowing the visualization of DOPr and MOPr at a subcellular resolution (Erbs et al., 2015). Although both receptors shared a similar distribution in some brain regions (e.g., basal ganglia), they appeared to be differentially expressed in several others (Erbs et al., 2015). While the DOPr-eGFP showed a high signal density in the external plexiform layer of the olfactory bulb, the anterior part of the basolateral amygdaloid nucleus, the cerebral cortex and parts of the brainstem (mainly the nucleus of origin of efferents of the vestibular nerve, reticulotegmental nucleus of the pons, pontine and external cuneate nuclei), the MOPr-mCherry seemed to be predominantly expressed in the anterior olfactory nucleus, the bed nucleus of the accessory olfactory tract, extended amygdala (intermediate part of the central amygdaloid nucleus and anterior dorsal part of the medial amygdaloid nucleus), dorsal midline and intralaminar thalamic nuclei (including the paratenial, rhomboid, xiphoid and centrolateral thalamic nuclei), hypothalamus (mainly the lateral hypothalamic area, as well as the medial parts of the medial- and supra-mammillary nuclei), epithalamus (MHb and *fasciculus retroflexus*) and parts of the brainstem (IPN, caudal part of the dorsal raphe nucleus, external part of the lateral parabrachial nucleus and parathrochlear nucleus) (Gardon et al., 2014; Erbs et al., 2015). In the spinal cord, MOPr-mCherry was mainly expressed in the superficial layers of the dorsal horn (principally lamina II), but fluorescent signals could also be detected in somas of all layers (Erbs et al., 2015). In dorsal root ganglia (DRG), both DOPr and MOPr were present in neurons with small-, medium-, and large-diameter somata (Erbs et al., 2015). However, while DOPr seemed to be more abundant in large diameter cells, MOPr appeared to be rather primarily expressed in small diameter neurons, which is in agreement with previous immunohistochemistry experiments performed in rodents (Rau et al., 2005; Scherrer et al., 2006; Wang et al., 2010).

By co-immunoprecipitation assays carried out using hippocampal tissues harvested from these dKI-FP mice, the authors also described the observation of DOPr/MOPr heteromers (Erbs et al., 2015). A few years later, heteromerization of endogenous MOPr-mCherry and DOPr-eGFP was further investigated by Derouiche et al. (2020). In this study, co-internalization of both receptors following stimulation with either DAMGO (MOPr agonist) or deltorphin II (DOPr agonist), but not SCN80, morphine or methadone was observed (Derouiche et al., 2020). Interestingly, the fate of the presumed DOPr-MOPr heteromers following ligand exposure resulted in their sorting to the lysosomal compartments (Derouiche et al., 2020). As recently reviewed in Degrandmaison et al. (2021), the DOPr displays several particularities regarding the regulation of its trafficking, including its preferential targeting to lysosomes following agonist-induced internalization, as opposed to most GPCRs, including MOPr and KOPr, which are typically recycled efficiently to the neuronal plasma membrane (Ko et al., 1999; Tsao and von Zastrow, 2000; Whistler et al., 2002; Tanowitz and von Zastrow, 2003; Tanowitz et al., 2008; Degrandmaison et al., 2021). Interestingly, this specific ligand-selective co-internalization and sorting of DOPr-MOPr heteromers to lysosomes thus suggests a DOPr-driven mechanism and might also represent a potential approach to fine-tune MOPr-mediated downstream signaling (Derouiche et al., 2020).

Grecksch et al. (2011) have investigated the role of MOPr phosphorylation on analgesic tolerance *in vivo* using a KI mouse expressing a mutant of the receptor in which the serine 375 is replaced by an alanine (MOPr^*S*375*A/S*375*A*^) (Figure 1E). In this study, the authors first observed that treatment of mice with escalating doses of morphine for 9 days strongly upregulated MOPr expression (Grecksch et al., 2011). Interestingly, this increase was not observed in saline- or etonitazene-treated animals (Grecksch et al., 2011). Since MOPr^*S*375*A/S*375*A*^ KI mice exhibited greater dose-dependent acute antinociceptive responses to morphine and fentanyl, phosphorylation of S375 was thus proposed to be involved in acute MOPr desensitization (Grecksch et al., 2011). These animals nevertheless still developed acute and chronic tolerance to morphine (Grecksch et al., 2011). Additional results obtained suggested that DAMGO- or etonitazene-induced tolerance required S375 phosphorylation, whereas morphine-mediated development of tolerance was S375 phosphorylation-independent (Grecksch et al., 2011).

More recently, a *N*-terminally HA epitope-tagged MOPr KI mouse line (HA-MOPr) has been developed by the same group using the clustered regulatory interspaced short palindromic repeats and its associated protein-9 (CRISPR/Cas9) technology (Figure 1F; Fritzwanker et al., 2021). Relying on small guide RNAs that direct the RNA-guided nuclease Cas9, this system enables the possibility of generating KO and KI mutant mice using a relatively fast and simple methodology (Gaj et al., 2013; Hsu et al., 2014). By performing mass spectrometry (MS) analyses of immunoprecipitated HA-MOPr from brain tissues, the authors have investigated the *in vivo C*-terminal phosphorylation patterns, as well as the presence of non-canonical isoforms of the MOPr (Fritzwanker et al., 2021). While at least 12 alternative MOPr splice variants have been suggested to exist in mice, only a few studies have attempted to validate the endogenous expression of such predicted isoforms at the protein level (Pasternak and Pan, 2013; Fritzwanker et al., 2021). Given that most of the MOPr isoforms are expected to harbor *C*-terminal mutations and/or truncations, the fusion of a small epitope-tag such as HA to the *N*-terminal avoids the risk of interfering with the detection of MOPr variants. The same rationale may also be applied for the study of *C*-terminally located phosphorylation events, indicating that the HA-MOPr KI mouse line presents a valid design for the study of intracellular signaling. However, although Fritzwanker et al. (2021) have been able to identify the canonical MOPr sequence by MS on brain lysates, the authors failed to detect a significant quantity of any of the alternate *C*-terminal isoforms proposed to arise from alternative splicing. Additional assays in which the canonical HA-MOPr have been depleted from the brain lysate samples by multiple successive rounds of immunoprecipitation resulted in the same conclusion (Fritzwanker et al., 2021). Moreover, the HA-MOPr KI mouse strain has facilitated high-resolution imaging of the receptor in the mouse brain, as well as the study of the molecular mechanisms related to agonist-induced phosphorylation (Fritzwanker et al., 2021). Using phosphosite-specific antibodies, the authors reported that high efficacy MOPr agonists such as methadone, fentanyl, sufentanil, and etonitazene promoted phosphorylation of Ser^375^, Thr^370^, Thr^376^, and Thr^379^ (Fritzwanker et al., 2021). Conversely, only Ser^375^ phosphorylation has been observed following treatment of the mice with either morphine or oxycodone, which are characterized as partial MOPr agonists (Fritzwanker et al., 2021). These results confirm previous *in vitro* observations reporting agonist-selective phosphorylation of the MOPr *C*-terminus.

Although still controversial and the current topic of heated debate, the recruitment of β-arrestin2 (βarr2) following morphine administration has been correlated with the deleterious side effects of opioids and reduced analgesia, suggesting that G protein-biased agonists might represent an improved class of analgesics (Bohn et al., 1999; Gillis et al., 2020). To investigate the concept of biased signaling *in vivo*, Ehrlich et al. (2019) have therefore designed an innovative tool. In this study, a novel MOPr-Venus-YFP (yellow FP) KI mouse line was generated by homologous recombination, resulting in the expression of the MOPr-Venus-YFP replacing the native murine receptor, in order to monitor the agonist-induced differential trafficking of the receptor (Figure 1G; Ehrlich et al., 2019). The authors selected the Venus-YFP since it represents the most versatile fluorophore compatible with resonance energy transfer (RET) biosensors and the most detectable in living cells (Nagai et al., 2002; Ehrlich et al., 2019). DRG neurons from MOPr-Venus-YFP KI mice were treated with 10 different MOPr agonists, including clinically prescribed opioids, classical peptides, and Gα_*i/o*_-biased agonists, to establish the specific trafficking profile induced by each compound (Table 1; Ehrlich et al., 2019). Interestingly, the authors observed that buprenorphine harbors a similar signature to the recently developed Gα_*i*_-biased drugs TRV 130 and PZM21 since it induced virtually no receptor redistribution or endosome translocation *in vivo* (Table 1) and remained largely insensitive to the overexpression of βarr2 and G protein-coupled receptor kinase 2 (GRK2) in the assays performed in HEK293 cells (Ehrlich et al., 2019). Given that buprenorphine is already a clinically safe effective analgesic, these results suggest a reassessment of its potential as an alternative to current opioid therapeutics (Ehrlich et al., 2019).

Since 2019, several independent research groups have generated MOPr-Cre KI mouse lines, thus allowing the precise targeting, visualization, and manipulation of MOPr-expressing neurons *in vivo* (Märtin et al., 2019; Bailly et al., 2020; Okunomiya et al., 2020; Zhang et al., 2020). In a first study carried out by Märtin et al. (2019), the authors successfully combined single-nucleus RNA sequencing (snRNAseq) of isolated *Oprm1* positive striatal projection neurons to create a spatiomolecular map that led to the identification and characterization of the molecular diversity of neuron subtypes in the striatum. To do so, the authors genetically labeled the striatal neurons expressing *Oprm1* through the generation of a MOPr-Cre KI mouse model (Figure 1H; Märtin et al., 2019). A T2A cleavable peptide sequence was inserted at the junction between the sequences encoding *Oprm1* and the Cre recombinase, allowing the release of the enzyme following translation of the MOPr-T2A-Cre fusion protein (Figure 1H; Märtin et al., 2019). This mouse strain was thereafter crossed with a Cre-dependent Ai14 reporter mouse line. Following Cre-mediated recombination, the resulting mice express robust tandem dimer Tomato (tdTomato) fluorescence in *Oprm1* positive striatal neurons allowing their visualization (Märtin et al., 2019). The spatiomolecular map provided by Märtin et al. (2019) established the differential signature of neuron subtypes and spatial markers for the identification of striatal subregions.

Designed by Okunomiya et al. (2020), a first inducible MOPr-CreER KI mouse strain has been developed by fusing the sequence encoding the Cre recombinase-modified estrogen receptor (CreER) with the T2A-like peptide sequence (Figure 1I). The resulting fusion was used to replace the STOP codon in the exon 4 of the *Oprm1* gene (Okunomiya et al., 2020). Using the estrogen receptor as a regulator of the Cre-recombinase protein allow its regulation through treatments with tamoxifen (Tm), thus creating an inducible genetic system (Okunomiya et al., 2020). The Tm-dependent expression of MOPr throughout the nervous system was mapped by breeding MOPr-CreER mice with the ROSA26 reporter mice, which conditionally express a HA-tagged bright monomeric teal fluorescent protein (mTFP1) following Cre-mediated recombination (Okunomiya et al., 2020). The authors observed that Tm-inducible MOPr-CreER-mediated recombination occurred in nearly all cells that would normally express the MOPr with a correlation of more than 94% between HA-mTFP1 fluorescent and *Oprm1* mRNA positive cells (Okunomiya et al., 2020). In the striatum, mTFP1-expressing cells exhibited a specific predominant localization in clusters presumably corresponding to striosomes (Okunomiya et al., 2020). Previously identified as MOPr-enriched compartments by immunohistochemistry (Arvidsson et al., 1995b; Kaneko et al., 1995; Mansour et al., 1995) and opioid binding autoradiography (Pert et al., 1976), these neural structures have been associated to diverse cognitive functions including reward prediction (Yoshizawa et al., 2018) and decision-making under conflict (Friedman et al., 2015). A similar strategy relying on the local injection of a Cre-dependent adeno-associated virus (AAV) vector in the striatum of MOPr-CreER mice has also been used to further validate the expression pattern of MOPr in the striosomes (Okunomiya et al., 2020). This latter approach combining viral vectors and MOPr-CreER mice thus represents a method to spatially restrict the transgene expression in MOPr-specific cell populations (Okunomiya et al., 2020). Given that the Tm-inducible MOPr-CreER mouse model allows a temporal control of the Cre-dependent recombination, such a lineage will also open the path to future studies focusing on the pre- and post-natal development of striatal compartments (Okunomiya et al., 2020).

Bailly et al. (2020) generated a KI mouse line, namely the MOPr-eGFP/Cre, in which the sequence encoding a functional eGFP/Cre recombinase fusion protein was inserted into exon 4 of *Oprm1*, upstream of, and in frame with, the STOP codon (Figure 1J). The generated MOPr-eGFP/Cre protein complex was expressed in the same neurons and cellular region as its wildtype homolog as transcription remained the same and localized in the studied region of the habenula (Bailly et al., 2020). In addition to the characterization of this novel mouse model, the authors demonstrated the possibility to manipulate specific MOPr-related circuitry by combining the MOPr-eGFP/Cre mouse line with optogenetics. Indeed, the precise activation of neurons in the VTA of MOPr-eGFP/Cre mice triggered place avoidance, supporting previous results described by Tan et al. (2012), Bailly et al. (2020). The use of optogenetics has also been applied to the study of chronic pain, as recently reported by Wei et al. (2021). In this article, the authors *shed light* on the implication of the dorsal (DH), but not the ventral (VH) hippocampus in the modulation of pain (Wei et al., 2021). More specifically, photostimulation of DH neurons for approximately 3 h was shown to relieve tactile allodynia in the spared nerve injury (SNI) neuropathic chronic pain model (Wei et al., 2021). Further experiments also suggested that the mechanism underlying the DH-mediated analgesic effects in SNI mice was dependent of *N*-Methyl-D-aspartate (NMDA) receptors, but also required the activation of MOPr (Wei et al., 2021). The development and use of optogenetic approaches will undoubtedly represent important tools for elucidating pain-related cellular mechanisms *in vivo*.

Another research group also took advantage of the Cre-mediated recombination system in order to selectively re-express MOPr in distinct neuronal population (Zhang et al., 2020). Zhang et al. (2020) first generated a KO mouse line by inserting a floxed STOP cassette between exon 1 and 2 of the *Oprm1* gene, thereby allowing re-expression of MOPr under the control of specific recombinases. Breeding of these mice with either Vglut2-Cre, Vgat-Cre, Lbx1-Cre, SNS-Cre or Nestin-Cre mice thus allowed the specific re-expression of MOPr in glutamatergic neurons, GABAergic neurons, dorsal spinal cord, small DRG neurons or in the nervous system, respectively (Figure 1K; Zhang et al., 2020). Using these newly developed conditional KI mice, the authors were able to characterize the expression, distribution pattern, as well as the contribution of each MOPr positive neuronal subpopulation in the mechanisms underlying analgesia in various pain models (Zhang et al., 2020). In a model of inflammatory pain, they observed that MOPr in Vglut2 + glutamatergic, but not GABAergic neurons mediated exogenous opioid-induced analgesia, whereas MOPr in GABAergic, but not Vglut2 + were rather involved in the endogenous opioid-induced analgesia (Zhang et al., 2020). At the spinal level, the analgesic effects of morphine mainly involve MOPr expressed in spinal glutamatergic neurons, in the context of acute pain (Zhang et al., 2020).

### δ-Opioid Receptor Knock-In Mouse Lines

Since its simultaneous cloning by two distinct groups in 1992, the DOPr has been recognized as a promising pharmacological target for the treatment of chronic pain, as well as various psychological and mood disorders (Evans et al., 1992; Kieffer et al., 1992; Gendron et al., 2016). In addition to the pain-alleviating properties reported in numerous chronic pain models (e.g., inflammatory, neuropathic, diabetic, and cancer), ligand-mediated activation of the DOPr has also been associated with anxiolytic, antidepressant, cardio- and neuroprotective effects (Kamei et al., 1997; Fraser et al., 2000; Brainin-Mattos et al., 2006; Holdridge and Cahill, 2007; Otis et al., 2011; He et al., 2013; Saitoh et al., 2013; Nozaki et al., 2014; Headrick et al., 2015). Most importantly, several studies have noted that its agonists produce considerably less undesired effects than most clinically prescribed opioids, thus rendering it an attractive alternative target to the MOPr for chronic pain management (Gallantine and Meert, 2005; Feng et al., 2006; Codd et al., 2009). However, the molecular and cellular mechanisms governing the trafficking and signaling of the DOPr are significantly distinct from most other GPCRs, including MOPr and KOPr, contributing to our limited understanding of its regulation (reviewed in Gendron et al., 2016; Degrandmaison et al., 2021). Consequently, the development of novel genetic tools is crucial in order to investigate its involvement in pathophysiological states and its potential as a therapeutic target.

In a groundbreaking study for both the opioids and GPCRs fields, Scherrer et al. (2006) designed the first opioid receptor KI mouse line by introducing the sequence encoding the eGFP into the exon 3 of the *Oprd1* mouse gene, in frame with the 5′-end of the STOP codon (Figure 1L). At that time, only one GFP-GPCR KI model had been generated, namely the human rhodopsin-GFP KI mouse (Chan et al., 2004). By combining homologous recombination and Cre recombinase treatment, the authors created a mouse line in which the WT DOPr is constitutively replaced by a fully functional DOPr-eGFP fusion (Figure 1L; Scherrer et al., 2006). Although a slight increase in the number of receptors and maximal GTPγS binding were observed, the potency of selective agonists such as SNC80, deltorphin II and the endogenous peptide Met-enkephalin remained unchanged in the KI mice (Table 1; Scherrer et al., 2006). Similarly, the affinity of the antagonist [^3^H]-naltrindole for the DOPr-eGFP was comparable to the WT receptor (Scherrer et al., 2006). While neuroanatomical analyses identified the detailed distribution of DOPr throughout the nervous system, real-time imaging in primary neurons and *in vivo* internalization assays have allowed the visualization of agonist-induced endocytocis of the eGFP-fused DOPr (Table 1; Scherrer et al., 2006). Altogether, these results indicate that the DOPr-eGFP mouse line represents a valid and useful tool to study the receptor localization and functions *in vivo*.

Since its generation, the DOPr-eGFP KI mouse model has been at the core center of numerous studies. Given the fluorescent property of this mouse strain, the distribution of the DOPr throughout the central nervous system and other discrete tissues has been obviously extensively investigated (Erbs et al., 2012, 2015; Rezaï et al., 2012; Guerrero-Alba et al., 2018). These mice have also proved useful to confirm previous *in vitro* results indicating that the DOPr is intracellularly sequestrated and subsequently targeted to lysosomal compartments following SNC80-induced internalization (Ko et al., 1999; Tsao and von Zastrow, 2000; Whistler et al., 2002; Wang et al., 2003; Pradhan et al., 2009, 2010). The studies carried out by Pradhan et al. (2009, 2010) also nicely illustrate the differential regulation of SNC80- or ARM390-mediated DOPr trafficking in a ligand-dependent manner. Although both compounds share similar binding, G-protein activation and analgesic properties, ARM390 did not trigger internalization of the receptors, as opposed to the rapid internalization induced by SNC80 (Pradhan et al., 2010). Such cellular findings are important since they explain behavioral observations denoting that the DOPr is unable to elicit antinociceptive responses to a second dose of the agonist SNC80, whereas a second injection of ARM390 produced potent antihyperalgesia, both 12 and 24 h after the first dose (Pradhan et al., 2009, 2010).

The modulation of the DOPr subcellular trafficking by various treatments and behavioral assays have also been widely investigated. For example, Dripps et al. (2020) recently described a significant increase in DOPr-eGFP expression in several forebrain regions, including the trigeminal nucleus caudalis and the trigeminal ganglia, following nitroglycerin-induced chronic migraine. Moreover, Bertran-Gonzalez et al. (2013) observed a learning-related translocation of DOPr-eGFP to the membrane of cholinergic interneurons of the NAc shell. These above-mentioned studies support the importance of the pioneering DOPr-eGFP KI mouse model as a tool to investigate this receptor *in vivo*.

More recently, two additional DOPr KI mouse lines have been generated, both relying on epitope-tagged receptors (Su et al., 2017; Degrandmaison et al., 2020). The first model, generated by Su et al. (2017), has been developed using the Transcription Activator-Like Effector Nucleases (TALEN) system. This approach allowing a one-step recombination uses a sequence-specific DNA-binding domain fused to a non-specific *Fok*I nuclease domain. Following binding to the predetermined DNA sequence, the enzyme will induce a double strand DNA break resulting in the engagement of DNA repair mechanisms, which in turn facilitates homologous recombination and the proper insertion of specific sequences within the targeted regions (Gaj et al., 2013; Joung and Sander, 2013; Su et al., 2017). In their study, the authors generated a mouse line expressing a *N*-terminally HA-tagged DOPr replacing the endogenous receptor (Figure 1M; Su et al., 2017). These mice were used to investigate the expression and distribution of the HA-DOPr in various brain sections and in the spinal cord (Su et al., 2017). The genetic design of this unique mouse strain also allows for the deletion of the receptor in specific tissues and/or cells following the removal of the floxed HA-DOPr sequence by a Cre recombinase (Su et al., 2017). This possibility has been further investigated by breeding HA-DOPr mice with Nestin-Cre mice in order to generate mice with a neural-specific DOPr KO (Su et al., 2017). These mouse lines represent powerful tools to study the molecular, cellular, and physiological functions of DOPr in distinct cell populations.

Using classical homologous recombination, our group has recently generated FLAG-DOPr KI mice (Degrandmaison et al., 2020). To create this lineage, we first designed a FLAG-DOPr-KO mouse strain containing the sequence encoding the FLAG epitope at the 5′-end of the *Oprd1* mouse gene (immediately after the START codon) and a translational STOP cassette flanked by two loxP sites inserted within the 5′-untranslated region (5′-UTR) (Degrandmaison et al., 2020). The presence of the STOP cassette disables the expression of FLAG-DOPr in all tissues, and by doing so, confers initial genotypic characteristics of a KO model (Degrandmaison et al., 2020). The breeding of these FLAG-DOPr KO mice with Zp3-Cre mice resulted in the excision of the floxed sequence which enables the expression of FLAG-tagged DOPr in all tissues that would normally express the WT DOPr (Figure 1N; Degrandmaison et al., 2020). An important feature of the FLAG-DOPr KO model resides in the possibility of generating conditional KI using distinct Cre recombinases allowing to rescue DOPr expression in a tissue- and/or cell-specific manner (discussed in section “Epitope-tagged G protein-coupled receptors knock-in mouse lines”) (Degrandmaison et al., 2020). The expression levels and distribution in the brain, as well as the functional characterization (i.e., G protein coupling, locomotor and pain behavioral studies) of the endogenous FLAG-DOPr demonstrated that FLAG-DOPr KI mice display similar behavioral and pharmacological properties as WT DOPr mice (Table 1; Degrandmaison et al., 2020). We used the unique properties of the FLAG-DOPr KI mice to reveal the first *in vivo* interactome of a GPCR (Degrandmaison et al., 2020). Indeed, proteomic analyses of the immunoprecipitated FLAG-DOPr from the forebrain of FLAG-DOPr KI mice allowed the identification of previously reported and, most importantly, novel endogenous DOPr-interacting proteins (Degrandmaison et al., 2020). Among the newly identified interactors, Rab10 has been further characterized and shown to be involved in the cell-surface targeting of the DOPr (Degrandmaison et al., 2020). Such an approach opened the path to exciting perspectives regarding the investigation of protein-protein interactions *in vivo* (further discussed in section “Epitope-tagged G protein-coupled receptors knock-in mouse lines”).

### κ-Opioid Receptor Knock-In Mouse Lines

As for the other OPr subtypes, activation of the KOPr has also been reported to elicit analgesia (Pathan and Williams, 2012). However, the wide range of adverse effects associated with its agonists, including dysphoria, aversion, stress-induced anxiety, sedation and psychotomimesis, has limited their clinical development for chronic pain-related treatment (Pfeiffer et al., 1986; Dykstra et al., 1987; Roth et al., 2002; Land et al., 2009). Actual research thus explores various strategies to limit these undesired physiological responses by focusing on the design of peripherally restricted KOPr agonists, G protein-biased agonists or drugs targeting several OPrs simultaneously (Paton et al., 2020). Nevertheless, the selective KOPr antagonist JNJ-67953964 or *Opra Kappa* (previously LY2456302) is currently under clinical development for the treatment of major depressive disorders (Reed et al., 2018; Jacobson et al., 2019). KOPr-targeted therapeutics also represent promising avenues for the management of other pathologies such as schizophrenia and drug addiction (Clark and Abi-Dargham, 2019). Although the first KOPr KO animal has been generated more than two decades ago, only two KI mouse lines, namely the KOPr-Cre and KOPr-tdT, have been developed until now (Cai et al., 2016; Chen et al., 2020). Considering the significant therapeutic potential of KOPr in numerous pathophysiological conditions, further research efforts focusing on this receptor are expected in the coming years.

Cai et al. (2016) generated a KOPr-Cre KI mouse line, representing the first developed OPr-Cre recombinase mouse strain. In these animals, the coding region of the *Oprk1* gene in exon 2 is replaced with the sequence encoding a Cre Recombinase (Figure 1O; Cai et al., 2016). Such a model allows for the conditional manipulation of cells specifically expressing KOPr (Cai et al., 2016). The authors further characterized the Cre-mediated recombination by breeding KOPr-Cre KI mice with mice displaying a Cre-dependent allele Rosa*^lsl tdTomato^* (Cai et al., 2016). Results indicated that KOPr is expressed in numerous cell types throughout the organism, including the cerebral cortex, NAc, DRG, striatum, heart, lung, and liver (Cai et al., 2016).

More recently, Chen et al. (2020) published three-dimensional (3D) images of the brain distribution of KOPr, a first for a GPCR, using a newly generated mouse line expressing the receptor fused to the fluorescent protein tdTomato (tdT) at its *C*-terminal extremity (Figure 1P). This novel mouse strain, henceforth referred to as KOPr-tdT, has been used to investigate the agonist-induced internalization of KOPr, as well as its neuroanatomical and cellular distribution (Table 1; Chen et al., 2020). To do so, the authors adapted the electrophoretic tissue clearing (ETC)-CLARITY method previously described by Kim et al. (2015) to generate 3-D images of KOPr brain distribution (Chen et al., 2020). The (ETC)-CLARITY clearing tissue approach, involving perfusion of animals with fixatives and acrylamide-based hydrogel and removal of lipids by detergents, allows the obtention of an optically transparent tissue while conserving its structural integrity (Kim et al., 2015; Chen et al., 2020). The elegantly displayed 3-D videos and brain images presented by Chen et al. (2020) suggest that KOPr is expressed in regions related to pain modulation, reward and aversion, as well as other areas for which a clear role has not yet been established such as the claustrum, dorsal endopiriform nucleus, lateral habenula and paraventricular nucleus of the thalamus. Although the specific structures were not identified, modest expression levels of KOPr were also observed in the spinal cord and DRGs (Chen et al., 2020). The optimized (ETC)-CLARITY tissue clearing method and the novel KOPr-tdT mouse line developed by the authors therefore represent useful tools for future investigations regarding the neuroanatomy of GPCRs, including OPr, as well as KOPr functions in various circuitries.

### Nociceptin/Orphanin FQ Receptor Knock-In Mouse Lines

Officially classified as a non-opioid member of the OPr family, the NOPr displays a unique pharmacological profile characterized by a low affinity for standard opioid peptides and antagonists, including the clinically approved drug naloxone (Pathan and Williams, 2012; Borsodi et al., 2019). Moreover, despite exhibiting 55–59% of sequence identity with the other three OPr subtypes, the NOPr is associated with dichotomous physiological effects regarding the modulation of pain transmission (Reinscheid et al., 1995; Grisel et al., 1996; Mogil et al., 1996a,b; Xu et al., 1996; Yamamoto et al., 1997; Mogil and Pasternak, 2001; Degrandmaison et al., 2021). Depending on several factors such as the dose, route of administration, type of pain stimulus, species or strains, agonists of the NOPr have been shown to induce both pro-nociceptive and analgesic actions (Reinscheid et al., 1995; Grisel et al., 1996; Mogil et al., 1996a,b; Xu et al., 1996; Yamamoto et al., 1997; Mogil and Pasternak, 2001; Donica et al., 2013). For example, while the spinal administration of its endogenous ligand nociceptin/orphanin FQ (N/OFQ) is mainly antinociceptive, intracerebroventricularly injected N/OFQ was found to block opioid-mediated analgesia, a physiological response frequently referred to as “anti-opioid activity” (Reinscheid et al., 1995; Grisel et al., 1996; Mogil et al., 1996a,b; Xu et al., 1996; Yamamoto et al., 1997; Mogil and Pasternak, 2001). These observations highlight the distinctively complex regulation of the NOPr and its close connection to the classical opioid system. As a result of its later discovery, the NOPr is the least well-characterized receptor as compared to the other OPr subtypes (Bunzow et al., 1994; Fukuda et al., 1994; Mollereau et al., 1994; Wang et al., 1994). Hence, the development of novel genetic tools is crucial to further improve our knowledge of the molecular and cellular mechanisms regulating this receptor, as well as its involvement in pain-, memory-, stress/anxiety- and drug reward-related pathways.

To our knowledge, the NOPr-eGFP and NOPr-eYFP KI mouse lines represent the only available KI animal models for this receptor (Ozawa et al., 2015; Mann et al., 2019). In both cases, using a similar approach as described for the generation of DOPr-eGFP and MOPr-mCherry (see sections “μ-opioid receptor knock-in mouse lines” and “δ-opioid receptor knock-in mouse lines”), the authors specifically inserted the sequence encoding the eGFP or eYFP into the *Oprl1* mouse gene, in frame and 5′ from the STOP codon, resulting in mouse strains expressing functional NOPr-FP *C*-terminal fusions replacing the native NOPr (Figures 1Q,R; Ozawa et al., 2015; Mann et al., 2019). While the NOPr-eGFP has been mainly used to study the *in vivo* distribution and agonist-induced phosphorylation of the receptor, primary culture of ventral midbrain neurons from NOPr-eYFP KI mice have been employed to visualize its internalization (Table 1; Ozawa et al., 2015; Mann et al., 2019). In a first study, Ozawa et al. (2015) have mapped the expression of the NOPr-eGFP in the spinal cord, DRG neurons and numerous brain regions involved in both pain (e.g., thalamus, MHb, vlPAG and locus coeruleus) and reward (e.g., NAc, VTA, MHb, amygdala, hippocampus and IPN), which supports a role for this receptor in such circuitries. While previous *in vitro* [^3^H]-N/OFQ radiography performed by Neal et al. (1999) in DRGs resulted in no binding, the presence of NOPr-eGFP in these cells is in agreement with *in situ* hybridization and electrophysiological experiments (Murali et al., 2012; Ozawa et al., 2015). Expression of NOPr in the spinal cord and DRG neurons is also consistent with the effects of intrathecally administered N/OFQ on nociception (Xu et al., 1996; Yamamoto et al., 1997; Ozawa et al., 2015). Given that supraspinal administration of N/OFQ can reverse the morphine-induced antinociceptive responses, it is interesting to note that the authors have identified cells co-expressing both NOPr and MOPr in various brain regions, as well as DRG neurons (Grisel et al., 1996; Ozawa et al., 2015). Further studies will however be needed to provide new insights on the complex interplay between these receptors.

In a following study, the authors analyzed the spinal distribution of NOPr in a chronic neuropathic pain model of spinal nerve ligation (SNL) (Ozawa et al., 2018). The results indicated that a decrease in the NOPr-eGFP fluorescent signal was observed in the spinal dorsal lamina I and II outer, regions recognized to mediate noxious heat stimuli, as well as in the L4 and L5 ipsilateral DRGs of mice that had undergone SNL (Ozawa et al., 2018). The capacity to visualize the NOPr-eGFP in discrete locations following the induction of pathophysiological states or treatments further reinforces the usefulness of FP-fused receptor KI mouse lines.

More recently, Mann et al. (2019) have investigated the differential agonist-induced NOPr phosphorylation in brains of NOPr-eGFP mice. Using newly developed phosphosite-specific antibodies and mass spectrometry analyses, the authors have identified the mouse residues Ser^343^, Ser^348^, Thr^359^, and Ser^360^ of the NOPr as phosphorylated *in vivo* following the intraperitoneal administration of the non-peptide full agonist AT-202 (Mann et al., 2019). Conversely, Ser^346^ and Ser^351^ appeared to be constitutively phosphorylated in the absence of a ligand, but an increase of phosphorylation was nevertheless observed following agonist treatment (Mann et al., 2019). The role of GRK2 and GRK3 in the AT-202-induced NOPr phosphorylation was also validated *in vivo* using compound 101, a selective GRK2/3 inhibitor (Mann et al., 2019). Most importantly, ligand-specific patterns of agonist-mediated phosphorylation and internalization have also been investigated (Mann et al., 2019). While NOPr agonists AT-202, Ro64-6198 and SCH221510 induced a significant internalization and phosphorylation at Ser^343^, Ser^348^, Thr^359^, and Ser^360^, receptors of the ventral midbrain neurons isolated from NOPr-eYFP mice exhibited only a slight internalization following stimulation with NNC 63-0532 (Table 1; Mann et al., 2019). On the other hand, MCOPPB administration induced strong NOPr internalization and phosphorylation at Ser^343^, but only a weak phosphorylation signal was detectable at Ser^348^, Thr^359^, and Ser^360^ (Mann et al., 2019). The complex pharmacology and various physiological responses attributed to the NOPr might therefore be explained by such agonist-selective differential phosphorylation, which would presumably influence subsequent signaling.

### Limitations, Perspectives and Future Directions

The arsenal of currently developed OPr KI mouse lines provides exciting perspectives regarding future studies investigating the molecular and cellular mechanisms underlying opioid physiology and associated disorders. As discussed below, the considerable number of available OPr KI mice should allow for the rapid development of additional unique models that could be pivotal for both the opioids and the GPCRs fields.

#### Knock-In Mouse Lines Harboring a G Protein-Coupled Receptors-Fluorescent Protein Fusion

Breeding of already existing KI mouse lines could represent a relatively simple strategy to investigate the complex interplay between OPr subtypes. As describe above, such an approach has already been used for the generation of DOPr-eGFP/MOPr-mCherry dKI mice (Figure 1D; Erbs et al., 2015). Similarly, the interplay between KOPr/DOPr or KOPr/NOPr could be investigated through the development of DOPr-eGFP/KOPr-tdT dKI and NOPr-eGFP/KOPr-tdT dKI mouse lineages, respectively. Furthermore, breeding of the existing MOPr-mCherry/DOPr-eGFP dKI mouse with a KI mouse harboring a KOPr fused to a complementary FP (e.g., mTagBFP2) could result in an interesting and unique triple KI model allowing for the simultaneous mapping of all three OPr subtypes (Kleeman et al., 2018). A plethora of possibilities also emerged when considering other KI mouse lines outside of the OPr family, as exemplified by the generation of a MOPr-mCherry/CX3CR1-eGFP dKI reporter mouse line, a model used to investigate microglial expression of MOPr in the brain and spine (Maduna et al., 2019).

Although KI mice designed with a GPCR-FP fusion have been and are still considerably useful to investigate many aspects of the receptor’s physiology, these lineages seem to be less appropriate for some particular applications. The main concern of these strains relies on the significant size of the fused FP (>25 kDa), which might be problematic for some analyses. For example, despite the undeniable advances provided by the DOPr-eGFP KI mouse model, the fusion of the FP to the *C*-terminal of the DOPr has raised controversy regarding its subcellular localization (Table 1; reviewed in Gendron et al., 2016). While a predominant intracellular localization of the DOPr under basal conditions has been reported in numerous studies (Pasquini et al., 1992; Arvidsson et al., 1995a; Cahill et al., 2001a,b; Wang and Pickel, 2001; Lucido et al., 2005; Gendron et al., 2006; Shiwarski et al., 2017a,b), high membrane expression of DOPr-eGFP has been described in tissues from KI mice (Table 1; Scherrer et al., 2006). Although Scherrer et al. (2006) confirmed that the DOPr-eGFP-induced signaling was similar to the WT DOPr in transfected HEK293 cells, another study reported that the addition of a GFP to either the *N*- or *C*-terminus of the DOPr significantly altered its subcellular distribution in PC12 cells (Wang et al., 2008). Ozawa et al. (2015) also noticed a progressive increase in the number of plasma membrane-localized receptors in WT, heterozygous, and homozygous NOPr-eGFP KI mice, respectively (Table 1). Alteration of the trafficking of other GPCRs, including the cannabinoid 1 receptor, muscarinic M4 receptor and β-adrenergic receptors, has also been described following the addition of an eGFP (McLean and Milligan, 2000; Madziva and Michael, 2001; McDonald et al., 2007). Altogether, these results suggest that the *N*-terminal fusion of an eGFP to a receptor might facilitate its maturation and transport along the biosynthetic pathway or perhaps increase its stability. Conversely, Wang et al. (2008) observed that the DOPr plasma membrane density remained unchanged following fusion of short epitope-tag sequences (e.g., HA or Myc) to its *N*-terminal extremity, indicating that the use of smaller tags might represent a more suitable strategy depending on the experiments to be performed. It is worth noting that the cell-surface targeting of the DOPr-eGFP in KI mice could still be increased under specific physiological conditions, in agreement with previous observations (Bertran-Gonzalez et al., 2013). Current progress in the development of smaller FPs, such as the miRFP670nano (∼17 kDa), could eventually represent appealing alternatives for the development of future tools allowing *in vivo* imaging of GPCRs (Oliinyk et al., 2019).

For specific experimental purposes, the generation of GPCR-FP KI mouse lines for *in vivo* fluorescence imaging might be ultimately replaced by newly developed technologies. For example, Esteoulle et al. (2020) have recently synthesized a bright fluorogenic near-infrared probe enabling the specific and background-free imaging of an endogenous GPCR in living mice. Absorption and emission of the dimeric probe in the near-infrared region optical window permits optimal *in vivo* imaging by minimizing the light scattering and absorption in blood and tissues, resulting in enhanced tissue penetration (Stolik et al., 2000; Hilderbrand and Weissleder, 2010; Yuan et al., 2013; Esteoulle et al., 2020). Using the oxytocin receptor as a prototype to develop this unprecedented whole animal fluorescence imaging method, the authors successfully labeled endogenous receptors, an achievement once considered as excessively challenging due to the low expression levels of GPCRs (Esteoulle et al., 2020). Given that this first-of-kind approach could presumably be transposed to other GPCRs, the use of such probes might eventually be applied for the study of OPr. Similarly, although it was not carried out in living animals, advances have also been recently denoted for the visualization of endogenous MOPr and DOPr in striatal cholinergic interneurons (Arttamangkul et al., 2021). Using NAI-A594, a ligand-directed labeling agent, the authors fluorescently labeled both OPr from live brain slices and concluded that MOPr and DOPr function independently, despite being localized in the same neurons (Arttamangkul et al., 2021).

#### Epitope-Tagged G Protein-Coupled Receptors Knock-In Mouse Lines

In some contexts, endogenously expressed epitope-tagged GPCRs represent an interesting alternative to a GPCR-FP fusion. As mentioned previously, peptide epitopes such as HA, FLAG or Myc might reduce the probability of altering the trafficking and function of the targeted receptor due to their small size ranging from 8 to 10 amino acids. This approach has been used to generate the HA-DOPr, FLAG-DOPr and HA-MOPr KI mice (Su et al., 2017; Degrandmaison et al., 2020; Fritzwanker et al., 2021). Since the *C*-terminal extremity of GPCRs represents a major site for post-translational modifications (PTM) and for the binding of effector proteins, fusion of the epitope-tag sequence to the *N*-terminal of the receptor has been carried out to prevent the potential gain and/or loss of interacting partners that could be mediated by an intracellular tag (Degrandmaison et al., 2021). Combination of these newly developed mouse lines with proteomics opened the path to a plethora of possibilities regarding the study of protein-protein interactions and PTMs. Akin to other GPCRs, OPr can undergo a wide range of constitutive and dynamic PTMs including glycosylation, palmitoylation, phosphorylation, and ubiquitination (Lemos Duarte and Devi, 2020; Degrandmaison et al., 2021). Since the fusion of the HA- or FLAG-sequence to the GPCR significantly facilitates its immunoprecipitation and enrichment from tissues, PTMs regulating OPr can be investigated *in vivo* using proteomic analyses or specific antibodies, as carried out by Fritzwanker et al. (2021). We have recently revealed the endogenous interactome of the DOPr by LC-MS/MS analyses performed on immunoprecipitated FLAG-DOPr from the forebrain of KI mice, thus leading to the identification of several potential DOPr-interacting proteins (Degrandmaison et al., 2020). Comparative interactome analyses between various regions of the brain, as well as other tissues including spinal cord and DRGs, could be pivotal in elucidating distinct DOPr functions throughout the nervous system. Similarly, comparison of the *in vivo* DOPr interactome following specific treatments or in various pain models (e.g., chronic morphine administration, inflammatory pain model) could provide leads for the study of the molecular mechanisms governing DOPr in pain-related pathways. Moreover, the complex interplay between DOPr and MOPr could also be investigated through the generation of a dKI mouse model, this time by breeding FLAG-DOPr mice with HA-MOPr mice. A compelling feature of such strain would reside in the possibility to study the protein–protein interactions of the receptors both simultaneously and independently. Indeed, distinct DOPr-, MOPr-, and DOPr/MOPr-interacting partners could be determined using a protocol combining sequential immunoprecipitations of FLAG- and HA-associated proteins, as well as LC-MS/MS analyses. An analogous strategy could be applied for the study of the KOPr- and NOPr-associated interactomes.

Similarly, such an approach could also be employed to investigate the connections existing between OPr and other physiological systems. For example, co-immunoprecipitation and BRET assays have suggested that the DOPr forms heteromers with the α_2*A*_-adrenergic receptor (α_2*A*_-AR) in HEK293 cells (Rios et al., 2004). Most interestingly, analgesic synergy between DOPr and α_2*A*_-AR has been observed following the spinal co-administration of some of their respective agonists (see Chabot-Doré et al., 2015 for a review). Combination therapy, in which the co-administered α_2*A*_-AR agonist acts as an adjuvant to opioids, represents a promising alternative with significant potential clinical impacts as it can conceivably improve the analgesic response while reducing opioid-related side effects (Chabot-Doré et al., 2015). To our knowledge, despite these biochemical and physiological observations, a direct interaction between DOPr and α_2*A*_-AR in native tissues has not been reported yet, and very little is known about the downstream signaling mechanisms participating in the synergistic opioid-adrenoreceptor axis (Chabot-Doré et al., 2015). Since Lu et al. (2009) have already generated a *N*-terminally HA-tagged α_2*A*_-AR KI mouse line (HA-α_2*A*_-AR), the interplay between these two systems could be investigated through the generation of a FLAG-DOPr/HA-α_2*A*_-AR dKI mouse.

Until now, visualization of FLAG-specific labeling in brain sections of FLAG-DOPr-KI mouse has been impaired by high fluorescence background signal (Degrandmaison et al., 2020). Non-specific binding of FLAG antibodies has been reported in brain tissues and cells by several research groups (Schäfer and Braun, 1995; Ferrando et al., 2015). In their study, Ferrando et al. (2015) generated numerous KI proteins harboring a 3xFLAG epitope that greatly improved their detection in all mice tissues except in the adult brain where non-specific labeling was still observed. An additional challenge resides in the low expression levels of DOPr, akin to other GPCRs, which certainly impacts on our capacity to visualize specific immunolabeling as compared to systems in which endogenous proteins are fundamentally highly abundant or overexpressed (e.g., using viral infections) (Degrandmaison et al., 2020). Using brain slices from a transgenic mouse strain expressing FLAG-MOPr targeted to catecholamine neurons, Arttamangkul et al. (2008) have successfully observed FLAG-specific staining of the receptors. However, one must keep in mind that the endogenous expression levels of DOPr and MOPr are different, and that the genetic approach used by the authors resulted in the expression of approximately twofold more FLAG-MOPr as compared to WT littermates, which might potentially facilitate the detection of the endogenous receptors (Arttamangkul et al., 2008). The generation of novel tools such as the FLAG-M5 monoclonal antibody (Sigma-Aldrich, Darmstadt, Germany, #F4042), which specifically recognizes *N*-terminal Methionine-FLAG fusion protein (*N*-term. Met-FLAG-protein), thus corresponding to the sequence harbored by the FLAG-DOPr-KI mouse, might represent an interesting alternative to overcome these challenges.

Conversely, the possibility to perform high-resolution imaging using HA-specific antibodies in neurons of HA-MOPr or HA-DOPr KI mice opens the path to upcoming investigations regarding their respective subcellular localization and intracellular trafficking. This approach also allows the study of the cellular redistribution of receptors *in vivo* in pathological conditions or following treatments. Akin to the DOPr (discussed in section “δ-opioid receptor knock-in mouse lines”), changes in the expression and/or subcellular localization of the MOPr in specific physiological conditions have been previously reported. In addition to the increase in MOPr expression following a 9 days treatment with escalating doses of morphine mentioned above (see section “μ-opioid receptor knock-in mouse lines”) (Grecksch et al., 2011), upregulation of MOPr has also been observed in corneal nerve fibers and trigeminal sensory neurons in a mouse model of inflammatory corneal pain (Joubert et al., 2020). In this study, topical ocular administration of DAMGO resulted in corneal hypersensitivity relief associated with inflammatory ocular pain (Joubert et al., 2020). On the other hand, downregulation of MOPr gene and protein expression has been described in L3-L5 DRGs removed from the ipsilateral side of rats with chronic inflammation of the knee joint, as well as in the synovium of adjuvant-induced monoarthritic rat knee joints (McDougall et al., 2004; Li et al., 2005). As another example, the fractionation and immunofluorescence assays performed by Mousa et al. (2013) revealed that MOPr displayed a predominant intracellular localization, and extensive co-localization with Rab7 in lysosome-associated membrane glycoprotein-1 (LAMP-I) positive perinuclear lysosomal compartments in DRG neurons of diabetic rats. By interfering with the Rab7-mediated lysosomal targeting of the receptors using intrathecally injected Rab7-siRNAs, the authors successfully restored the plasma membrane density of MOPr, as well as opioid responsiveness toward better pain relief in an animal model of diabetic neuropathic pain (Mousa et al., 2013).

Furthermore, the genetic design used to create the above-mentioned FLAG-DOPr KO mouse line (see section “δ-opioid receptor knock-in mouse lines”) enables the generation of conditional KI animals (Degrandmaison et al., 2020). Indeed, the specific Cre-driven excision of the translational STOP cassette can allow the rescue of DOPr expression in target tissue and/or cells. As a proof of concept, we used the recombinant adeno-associated virus rAAV2/9-CBA-Cre-GFP (Abdallah et al., 2018), which predominantly targets lumbar DRGs following intrathecal administration, to specifically re-express the receptor in these neurons (Degrandmaison et al., 2020). In a CFA-induced chronic pain model, our results indicated that the antihyperalgesic effects of deltorphin II, a DOPr specific agonist, were partially reinstated 6 weeks post viral infection, thereby supporting a role for DOPr localized on primary afferents in the control of pain induced by a thermal stimulus (Degrandmaison et al., 2020). The FLAG-DOPr-KO mouse therefore represents a powerful genetic tool to decipher the roles of the DOPr in specific targeted regions.

#### Future Directions

The constant progress in the field of genome editing led to the development of numerous revolutionary tools including the CRISPR/Cas9 technology. As already described for the generation of the HA-MOPr KI mouse line (see section “μ-opioid receptor knock-in mouse lines”), this approach enables the possibility of generating KO and KI mutant mice by deleting, inserting or modifying a specific targeted gene (Hsu et al., 2014; Hall et al., 2018). In an attempt to guide future pharmacological treatments toward precision medicine, a tool such as CRISPR/Cas9 will undoubtedly potentiate the generation of novel mouse lines harboring mutations representative of identified SNPs, as described above for the MOPr-A118G, or other relevant mutations that would better our understanding of the molecular mechanisms related to opioid physiology (Mague et al., 2009). Possible involvement of the DOPr-F27C, KOPr-D374N or additional MOPr variants in different pathophysiological conditions could also be studied *in vivo* using the CRISPR/Cas9 technology (Gelernter and Kranzler, 2000). Moreover, as discussed for the MOPr-Cre strain (see section “μ-opioid receptor knock-in mouse lines”), combination of KI mouse lines with other emerging cutting-edge technologies, such as opto- and chemo-genetics, is expected to play a significant role in providing new insights on opioid physiology (Gillis et al., 2020). Members of the OPr family have been established once again as leading-edge receptors for the development of novel tools, as demonstrated by the recent design of the light-activable MOPr chimera “Opto-MOR” and the unique KOPr-DREADD (*Designer Receptor Exclusively Activated by Designer Drug*) chemogenetic system allowing the *in vivo* modulation of neuronal activity (Siuda et al., 2015; Vardy et al., 2015).

Recent advances in the GPCR field have also contributed to the improvement and development of novel technologies enabling a better understanding of their complex physiology. After several years of challenging optimization, the use of Resonance Energy Transfer (RET)-based biosensors in living organisms has finally been successful (van Unen et al., 2015; Kono et al., 2017). In their study, Kono et al. (2017) described the generation of a genetically engineered mouse used for the *in vivo* real-time imaging of the sphingosine-1-phosphate receptor 1 (SIP_1_) signaling by reporting the interaction with βarr2. Upon receptor activation, complementation of the engineered firefly split luciferase fragments produces an active enzyme complex that, in the presence of ATP and luciferin, generates light detectable by bioluminescence imaging (Kono et al., 2017). If transposed to the study of OPr, such bioluminescent mouse models might have a significant impact on the development of novel opioid therapeutics, as well as on our understanding of the specific downstream signaling pathways activated by distinct agonists.

## Conclusion

Given their substantial therapeutic importance, OPr have been widely used as prototypic GPCRs for the development of several novel *in vivo* genetic tools. Indeed, OPr KI mouse lines have been the basis of important advances in the GPCR field, including the generation of the second GFP-GPCR fusion KI mouse model (Scherrer et al., 2006), the first 3-D brain images to investigate the endogenous distribution of a GPCR (Chen et al., 2020), and the first *in vivo* interactome of a GPCR (Degrandmaison et al., 2020). The combination of the recently developed OPr-Cre KI mice, as well as the “KOPr-DREADD” and the light-activable “Opto-MOR” with opto- and chemo-genetic approaches opens the path to the visualization and, more importantly, the direct *in vivo* manipulation of specific neurons and targeted circuitries (Siuda et al., 2015; Vardy et al., 2015; Cai et al., 2016; Märtin et al., 2019; Bailly et al., 2020; Okunomiya et al., 2020). In addition to the above-mentioned perspectives, the myriad of exciting possibilities regarding the generation of unique mouse strains is further multiplied when considering the breeding of KI and KO models. Finally, the approaches developed to investigate the functions of OPr *in vivo* could also be pivotal for the study of other GPCRs, as the genetic strategies can be conceivably transposed.

## Author Contributions

JD, SR-H, J-LP, and LG wrote the manuscript. All the authors contributed to the article and approved the submitted version.

## Conflict of Interest

The authors declare that the research was conducted in the absence of any commercial or financial relationships that could be construed as a potential conflict of interest.

## Publisher’s Note

All claims expressed in this article are solely those of the authors and do not necessarily represent those of their affiliated organizations, or those of the publisher, the editors and the reviewers. Any product that may be evaluated in this article, or claim that may be made by its manufacturer, is not guaranteed or endorsed by the publisher.

## Abbreviations

α_2*a*_-AR: alpha-2a adrenergic receptor
AAV: adeno-associated virus
β arr2: beta-arrestin 2
BRET: bioluminescence resonance energy transfer
CFA: complete Freund’s adjuvant
CPu: caudate putamen
CreER: Cre recombinase-modified estrogen receptor
CRISPR/Cas9: clustered regulatory interspaced short palindromic repeats and its associated protein-9
DAMGO: [D-Ala^2^, *N*-Me-Phe^4^, Gly^5^-ol]-enkephalin
DH: dorsal hippocampus
dKI: double knock-in
DOPr: delta-opioid receptor
DREADD: designer receptor exclusively activated by designer drug
DRG: dorsal root ganglion
eGFP: enhanced green fluorescent protein
(ETC)-CLARITY: electrophoretic tissue clearing-CLARITY
FP: fluorescent protein
FRT: flippase recognition target
GPCR: G protein-coupled receptor
GRK2: G protein-coupled receptor kinase 2
HA: hemagglutinin
HEK293: human embryonic kidney cells
IPN: interpeduncular nucleus
KI: knock-in
KO: knock-out
KOPr: kappa-opioid receptor
LAMP-I: lysosome-associated membrane glycoprotein-1
MHb: medial habenula
MOPr: mu-opioid receptor
MS: mass spectrometry
mTagBFP2: monomeric Tag blue fluorescent protein
mTFP1: monomeric teal fluorescent protein 1
N/OFQ: nociceptin/orphanin FQ
NAc: nucleus accumbens
NMDA: *N*-Methyl-D-aspartate receptor
NOPr: nociceptin/orphanin FQ receptor
OPr: opioid peptide receptors
PAG: periaqueductal gray
PTM: post-translational modification
RET: resonance energy transfer
S1P1: sphingosine-1-phosphate receptor 1
SNC80: 4-[(R)-[(2S,5R)-4-allyl-2,5-dimethylpiperazin-1-yl](3-methoxypheny) methyl]-*N,N*-diethylbenzamide
SNI: spared nerve injury
SNL: spinal nerve ligation
SNP: single nucleotide polymorphism
snRNAseq: single-nucleus RNA sequencing
T2A: *Thosea asigna* virus 2A-like peptide
TALEN: transcription activator-like effector nuclease
tdTomato: tandem dimer Tomato
Tm: tamoxifen
VH: ventral hippocampus
VTA: ventral tegmental area
WT: wildtype
YFP: yellow fluorescent protein.
